# The Prevalence and Replication Capacity of a Tibetan Dominant HBV Strain, C/D Recombinant

**DOI:** 10.1155/2017/8415907

**Published:** 2017-06-21

**Authors:** Lingyao Du, Menghan Liu, Miao Liu, Taoyou Zhou, Xing Cheng, Cong Liu, Hong Tang

**Affiliations:** ^1^Center of Infectious Diseases, West China Hospital of Sichuan University, Chengdu 610041, China; ^2^Department of Science and Technology, West China Hospital of Sichuan University, Chengdu 610041, China

## Abstract

This study aimed to estimate the distribution of hepatitis B virus (HBV) C/D recombinant in Han and Tibet patients with chronic hepatitis B (CHB) and then learn such strain's replication capacity in vivo. A total of 331 serum samples were collected from Han outpatients from Sichuan Province and Tibetan outpatients from Tibet. Viral genotypes in these samples were identified. An HBV replicative plasmid of C/D recombinant was constructed with selected genome. Sequentially, HBV replicative mouse models were established and the replication capacity of the viral strain was studied in vivo. In the 314 Han patients, 66% (207) were infected by genotype B strain while 31% (96) were by genotype C strain. Only 1% (3) were by C/D recombinant. In the 17 Tibetan patients, 41% (7) were by genotype D and 35% (6) by C/D recombinant. A plasmid with 1.3 copies of C/D recombinant genome was constructed. And its replication intermediates were found at similar levels to that of genotype D strain. Thus, C/D recombinant, the dominant viral strain in Tibet, was rather rare in the genotype B predominated Han patients from Sichuan Province. And the C/D recombinant replicated at a similar level to viral strain of genotype D in vivo.

## 1. Introduction

Decades ago, HBV were subtyped by the amino acid sequences of their surface antigen (HBsAg) [1]. Along with the development of direct sequencing, new method emerged for HBV classification. Phylogenetic analysis of the entire genome has been used for HBV genotyping. Though time consuming and expensive, it is generally accepted as the golden standard [2].

Nine HBV genotypes ranging from A to I and a putative 10th genotype J have been identified by now [3–7]. The intergroup diversity of each genotype is greater than 7.5% in genomic nucleotide [8]. Viral strains from different genotypes represent unique biological characteristics; these biological characteristics result in various clinical manifestations of the infected patients. Current studies have suggested a causal relationship between HBV genotypes and the spectrum of HBV related diseases, leading us to further investigation in viral genotypes [9–14].

Subgenotypes are evolutionary swarms in genotypes with genomic diversity between 4% and 7.5% [14]. However, under such rule, some recombinant strains were misclassified into subgenotypes. Currently, some scientists suggested that the intragenotypic nucleotide divergence between 4% and 7.5% was not the best prerequisite to identify subgenotypes [15, 16]. Even there is traces of recombination in some subgenotypes; recombinant strains should be classified into an independent group when evidence of combination was confirmed and the genetic divergence fell into the classical definition of subgenotypes [17]. Based on that, more than 30 recombinant strains have been reported [18]. The recombination regions were always the essential functioning regions affecting viral biological characteristics, and these characteristics lead to various manifestations in patients [19, 20].

Geographic environment and genetic background contribute to the development of genotypes, subgenotypes, and recombinants [21]. In Tibet, the summit of Asian continent, HBV infection is highly prevalent in patients. In 2002, local doctors conducted a viral sequence analysis with over 1000 serum samples from asymptomatic chronic HBV carriers. The sequencing result showed highly homologous sequences, implying the existence of a dominant strain. Phylogenetic analysis in its S gene identified it as genotype D, but it lacked the typical nucleotide loss in nt2855-2887. Full genome analysis classified it into genotype C. Such results strongly suggested that the dominant strain in Tibet is a new recombination. Later, a representative genome was acquired. Full genome as well as each open reading frame was analyzed with the 23 standard sequences from genotype A to F in NCBI. The result showed a region around nt50-1540 covering preS2/S region and part of the P region from genotype D was integrated into genotype C to form the recombinant viral strain [22].

Reports on C/D recombinant followed the previous finding. A study in Uygur from Shinkiang found that the C/D recombinant was epidemic in 109 chronic HBV carries, with a ratio of 29.4% (*N* = 32) [23]. Another study in 2009 confirmed that the C/D recombinant was dominant in both Shinkiang and Tibet [24]. However, the distribution of the viral strain in Han nationality remains unknown. And relationship between viral biological characteristics and the minority population susceptibility in west China needs further investigation.

Replicative HBV plasmid is applicable to study the viral biological characteristics. Such plasmid can replicate effectively both in vitro and in vivo. It contains 1.3 copies of HBV genome with 4.1 Kb in length in its multiple cloning sites. Besides 3.2 Kb of the HBV genome, a 0.9 Kb of the repeating sequence from upstream Enhance I and X promoter to downstream Poly A is integrated. Thus the plasmid usually named pHBV4.1 [25].

In this study, we aimed to roughly estimate the distribution of HBV C/D recombinant in Han nationalities in Sichuan Province, West China, as well as Tibetan patients in Tibet. We also aimed to first identify the special viral strain and then to extract it to construct a replicative plasmid pHBV4.1(C/D). By doing so, we hoped to understand the biological characteristics of such special HBV strain.

## 2. Results

### 2.1. Distribution of C/D Recombinant Strain in Serum Samples from Different Groups

In this study, we collected a total of 331 serum samples, and five genotypes were identified. 210 samples were identified as genotype B. 97 samples were identified as genotype C, and 7 samples were identified as genotype D. B/C recombinant strain and C/D recombinant strain were found in 8 and 9 samples, respectively.

Based on the patients' ethnic background, the samples were divided into two groups: the Han group and the Tibetan group. In the Han group: 314 serum samples were collected from Han CHB patients from Sichuan Province; and 4 viral strains were detected; genotype B strain infected 207 (66%) patients, genotype C strain infected 96 (31%) patients, B/C recombinant strain infected 8 (2%) patients, and C/D recombinant strain infected only 3 (1%) patients. In the Tibetan group: 17 serum samples were collected from Tibetan CHB patients from Tibet, and also 4 viral strains were detected; genotype B strain infected 3 (18%) patients, genotype C strain infected 1 (6%) patient, genotype D strain infected 7 (41%) patients, and C/D recombinant strain infected 6 (35%) patients. Moreover, the viral load, composition of BCP mutations and liver stiffness also showed significant differences in the Han group and the Tibetan group. Composition of each virus strain in these two groups and other information were shown in Table 1.

To learn the unique clinical manifestation or virological features of C/D recombinant, these data in C/D recombinant infected patients were analyzed and compared with that of all patients. As mentioned before, 9 patients were confirmed to be infected with HBV C/D recombinant in total. It turned out that alcohol-abuse rate was higher in C/D recombinant infected patients as well as the occurrence rate of BCP A1762T/G1764A double mutation. The detailed clinical information was listed in Table 2.

### 2.2. Construction of HBV Replicative Plasmid pHBV4.1(C/D)

**Figure 1:**
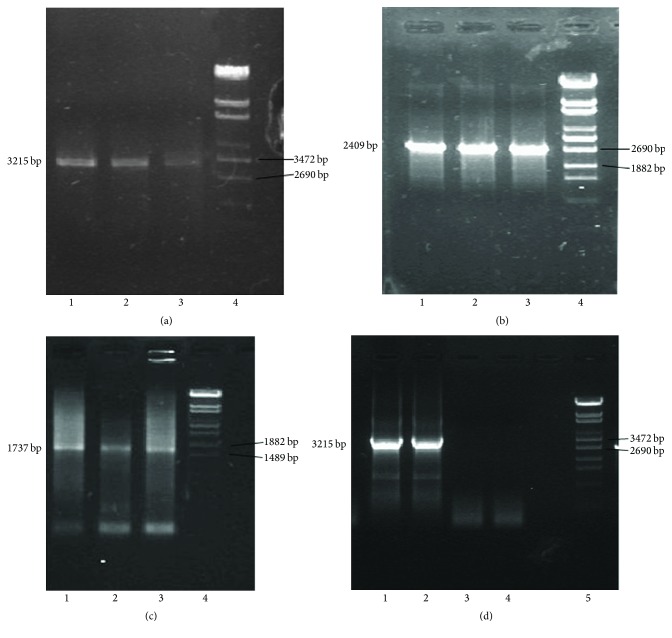
Gel bands during the construction procedures of HBV replicative plasmid of C/D recombinant. (a) Amplification of HBV full genome with templates extracted from serum samples. Lines 1–3 were amplification products. Line 4 was *λ*DNA/EcoT14 digested marker. (b) Amplification of HBV fragment of 2.4 Kb with amplified and ligated HBV genomic DNA as templates. Lines 1–3 were amplification products. Line 4 was *λ*DNA/EcoT14 digested marker. (c) Amplification of HBV fragment of 1.7 Kb with amplified and ligated HBV genomic DNA as templates. Lines 1–3 were amplification products. Line 4 was *λ*DNA/EcoT14 digested marker. (d) Amplification of HBV full genome with newly constructed replicative plasmid. The plasmid was derived from ligation of 1.7 Kb and 2.4 Kb fragments and vector pUC18. After transfection and selection, positive DH5*α* clones were acquired for plasmid extraction and full viral genome amplification was conducted for verification. Lines 1 and 2 were amplification products with selected plasmid as template. Lines 3 and 4 were amplification products with only vector pUC18 as templates. Line 5 was *λ*DNA/EcoT14 digested marker.

**Figure 2:**
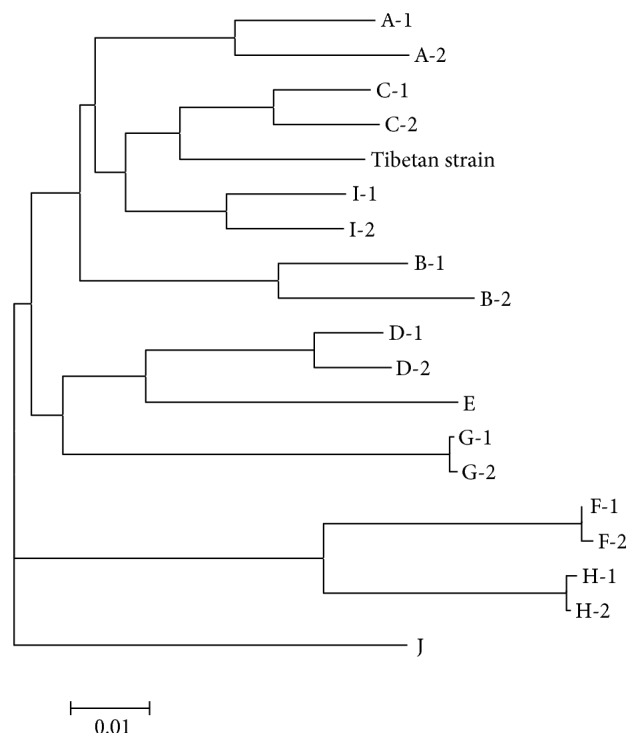
Phylogenetic analysis of the selected Tibetan viral strain. Reference Sequences from NCBI: genotype A: A-1(AM282986.1) and A-2(JN182318.1); genotype B: B-1(AB368295.1) and B-2(EF494382.1); genotype C: C-1(AB368296.1) and C-2(AB368297.1); genotype D: D-1(AM422939.1) and D-2(AB554024.1); genotype E: E(FN594748.1); genotype F: F-1(AB036909.1) and F-2(AB036907.1); genotype G: G-1(AP007264.1) and G-2(AB625342.1); genotype H: H-1(AB275308.1) and H-2(AB179747.1); genotype I: I-1(FJ023659.1) and I-2(FJ023664.1); genotype J: J(AB486012.1). Full HBV genomic sequences were applied for phylogenetic analysis.

**Figure 3:**
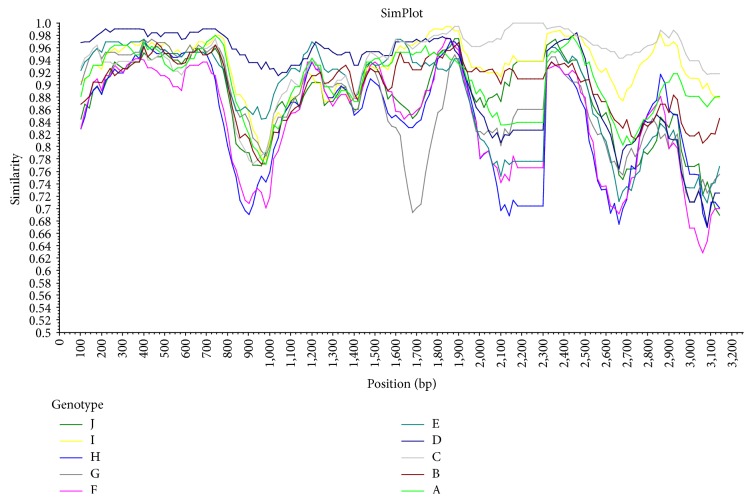
Recombination analysis of the selected Tibetan viral strain. Phylogenetic analysis data were also used as alignment data. The sequence similarity of the selected Tibetan strain to viral strains of all ten genotypes from A to J was analyzed. The position of nucleotide bases was shown in the abscissa and the similarity of the selected Tibetan strain to reference strains was shown in the ordinate. The numbering of HBV genome started from the restriction site of EcoR I downstream of preS2 initiation codon. Each curve represented one genotype and it showed the variation of sequence similarity between the selected Tibetan strain and the chosen reference sequence at each base site in the full genome. As shown, the dark blue curve, which was at the top left, implied that the fragment (nt1-1480) of the Tibetan HBV genome covering preS2/S region and X region had the highest similarity to genotype D. And the light grey curve, which was at the top right, implied that the rest of the genome had the highest similarity to genotype C.

One Tibetan serum sample which was infected by C/D recombinant strain showed high viral load. It was selected for DNA extraction and was then used as PCR template. With a specially designed primer pair, HBV full genomic DNA was acquired. Sequentially two fragments of 1.7 Kb and 2.4 Kb were successfully acquired, ligated, and inserted. Then one germ of clone containing newly constructed plasmid was picked out for confirmation. The result was that a fragment about 4.1 Kb could be digested out from the plasmid and full HBV genomic DNA about 3.2 Kb could be amplified from the recombinant plasmid for sequence confirmation. The related gel bands were showed in Figure 1. Amplification product was sequenced in SinoGenoMax Co., Ltd. (Beijing). The numbering of the HBV genome started with TTCC, namely, the restriction site of EcoR I (GGAATTC), downstream of the initiation codon (ATG) of preS2 region. In some strains with mutations, it started with CTCC. Phylogenetic analysis showed the sequence belonged to a branch next to genotype C (Figure 2). In the subsequent recombination analysis, a fragment (nt1-1480) covering preS2/S region and X region of the selected viral strain showed higher similarity to reference sequence of genotype D than the similarity to reference sequences of other genotypes including genotype C (Figure 3). This proved that the newly constructed plasmid was truly derived from the C/D recombinant.

### 2.3. Establishment of the Hydrodynamic HBV Replicative Mouse Model with pHBV4.1(C/D) and Study of Its Replication Capacity

Two groups of hydrodynamic HBV replicative mouse models were established with pHBV4.1(D) and pHBV4.1(C/D). The mouse model of pHBV4.1(D) was established and verified in our lab previously. It was confirmed to be efficient for learning HBV replication, transcription, and replication. Thus the replication capacity of pHBV4.1(C/D) could be studied in a similar model established according to the same procedures. With the same primer pair and reaction condition, full HBV genomic DNA in mouse serum was amplified. Then the amplified products were sequenced again. After BLAST, the relevant HBV sequences with an identity over 97% all belonged to C/D recombinant. The result was the same as the sequencing result after plasmid was successfully constructed.

**Figure 4:**
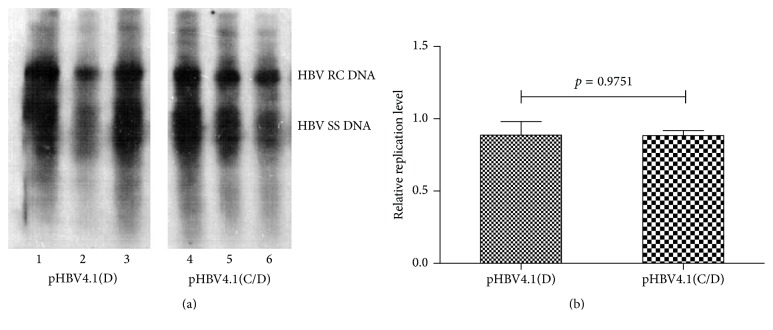
HBV replication intermediates in mouse models established with pHBV4.1(D) and pHBV(C/D). Mice were injected hydrodynamically with 10 *μ*g pHBV4.1(D) orpHBV4.1(C/D) and mouse livers were harvested 3 days after modeling. HBV replication intermediates in the liver were detected through DNA (Southern) filter hybridization (a). The image of DNA (Southern) filter hybridization was imported into Quantity One (Bio-Rad Laboratories, Life Science) and analyzed. The module of Volume Rect Tool was applied to generate rectangles covering all the bands in each lane and density of delineated bands was qualified and compared (b). The two plasmids possessed similar replication capacity in vivo.

Viral replication intermediates were isolated according to previously published method [26]. And with introduction of DNase digestion during the procedures, the input plasmid DNA was removed and the isolated nucleotides were purified replication intermediates. Three independent experiments were carried out and totally six mice were modeled in each experiment: 3 in the pHBV4.1(D) group and 3 in the pHBV4.1(C/D) group. Results showed that the HBV replicative intermediates could be detected by DNA filter hybridization in each group. The semiquantitative data captured by Quantity One showed that pHBV4.1(C/D) possessed similar replication capacity of pHBV4.1(D), demonstrating no significant differences (0.87 versus 1.00 *p* = 0.9751). It implied that newly constructed plasmid could replicate effectively and stably in mouse hepatocytes. Viral strain of C/D recombinant strain presented similar replication capacity as viral strain of genotype D (Figure 4).

## 3. Discussion

Geographically, HBV genotypic distribution in China is that genotype C is predominant in the north while genotype B is prevalent in the south. We found such genotypic distribution consistent in Han patients from Sichuan Province of Southwest China. The predominant strains in Sichuan Province were genotypes B and C, and genotype B was the majority. In the 314 Sichuan Han patients, only 3 patients were confirmed to be with C/D recombinant strain infection. But when it came to Tibetan patients, the situation was quite different. From the limited serum samples collected from Tibetan patients enrolled in the study, genotype D and C/D recombinant strains were dominant. There were 76% (*N* = 13) of Tibetan patients infected by these two viral strains, and 46% (*N* = 6) were confirmed to be infected by C/D recombinant strain. The genotype D strain seemed to infect more patients (*N* = 7) than C/D recombinant in the study. This result is different from the previous report [24]. The reason could be that all C/D recombinant strain shared the same sequence similar to the S region of genotype D viral strain. So when S gene sequencing was used for genotype identification, C/D recombinants could be mistaken as genotype D. Moreover, there was an isolate of HBV C/D recombinant with a deficiency of nucleotides between nt2853 and nt2855. And a repeating sequence of seven nucleotides (GCATGGG) located upstream and downstream of the lack, respectively. Such location and genomic structure perfectly mimic that of the genotype D viral strain which located between nt2855 and nt2887. This strain could also be mistaken as genotype D. Our results demonstrated that C/D recombinant of the HBV viral strain was a predominant HBV strain in Tibetan patients from Tibet and rarely seen in Han patients from Southwest China.

In 1999, Mayerat et al. theorized that HBV genetic divergence would affect the HBV related diseases' spectrum. Mayerat et al. not only proved that genotype A virus was related to chronic infection, but also for the first time related the genotype D virus to acute transformation of the disease [9]. This study brought attention to the correlation between HBV genetic divergence and clinical characteristics in patients and marked the start of various studies on this topic [10–13]. Various studies later all confirmed that viral genetic divergence could lead to the variation in clinical manifestation and prognosis of patients. However, current studies about C/D recombinant mainly focused on sequence study, distribution, and some other epidemiological characteristics. Data about the recombinant's biological characteristics is lacking, not to mention the clinical features of those infected patients. An effectively replicative plasmid would be helpful to solve the problem. In our study, the HBV replicative plasmid of C/D recombinant strain was successfully constructed. Thus HBV replicative animal models of this strain could be established for studies on viral replication, pathogenesis, and drug resistance afterwards.

Previously, the plasmid with 1.3 copies of HBV genome had been proved to replicate and transcribe stably as the natural HBV in hepatocytes in both transgenic mice and regular BALB/C mice [25, 26]. It is promising that the newly constructed plasmid with the same structure would replicate efficiently. And It showed similar replication capacity to pHBV4.1(D). For the first time, the replicative characteristics of the HBV C/D recombinant were studied. We adopted the genotype D strain as the control to assess the C/D recombinant not only for the proved stable replicative ability of genotype D, but also for its contribution of a recombinant region for the C/D recombinant. The fact that no significant difference was found may be resulting from the same S region and serotype (ayw) of the two strains. But learning the differences of HBV C/D recombinant from genotype D strain could still be a good start of further investigations.

The unique genome of HBV C/D recombinant was suggested to influence the patients' manifestation. Previous study also revealed that C/D recombinant exhibited higher frequency with HBeAg positive, high level of HBV DNA, and BCP A1762T/G1764A double mutation [27]. In our study, we found similar result in BCP mutation. The composition of BCP mutations showed significant difference between C/D recombinant group and the entire group (Fisher, *p* = 0.03049). The percentage of A1762T/G1764A double mutation in C/D recombinant was especially higher than the entire group (44.44% versus 16.01%). However, we did not observe any differences in the HBeAg positive frequency (*p* = 0.089) and HBV DNA load (*p* = 0.26) between C/D recombinant group and the entire groups. The samples size might account for this and further study remains to proceed.

Moreover, according to a previous study in Fujian province, China, genotype C, genotype D, and their recombinant were identified in patients with HBV associated hepatocellular carcinoma (HBV-HCC). Though the pathogenesis of C/D recombinant remains to be clarified and clinical manifestations of those infected patients need more data, we could still make a sidewise approach through genotype C and D strains. Two large scale clinical trials in Hong Kong and Taiwan confirmed that patients infected by genotype C strain encounter a higher risk of HBV-HCC genesis. And two studies in India and Iran showed that genotype D strain infection was related to higher histological inflammation and higher risk of HBV-HCC genesis [10, 11, 13]. Therefore, the C/D recombinant may have similar pathogenicity to these two strains (genotype C and genotype D). More investigations should be implemented to verify it.

With the use of the replicative plasmid, the basic mechanism of clinical manifestations, virological features, and viral pathogenicity could be clarified. Furthermore, C/D recombinant should have included two subrecombinants, namely, C/D1 and C/D2. The recombinant region in C/D1 strain covered preS2/S region (nt10-799), while C/D2 strain had a recombinant region from preS2/S to X regions (nt10-1499) [16]. In our study, the selected viral strain was more similar to C/D2 strain. Zhou et al. found that C/D1 strain infected patients would encounter lower serum bilirubin and lower frequency of G1896A mutation compared to C/D2 strain infected ones [24]. In the future, deep analysis into the C/D recombinant should be expected.

## 4. Materials and Methods

### 4.1. Study Materials

Serum samples were collected from Han and Tibetan outpatients consulted in West China Hospital of Sichuan University: 314 samples were from Han outpatients from Sichuan Province and 17 samples were from Tibetan outpatients from Tibet. The HBV replicative plasmid pHBV4.1(D) and the vector plasmid pUC18 were reserved in our lab. Competent bacterium* E. coli* DH5*α* was purchased from Takara Bio(Dalian) as well as LATaq PCR Amplification Kit, restriction endonuclease Hind III, Pst I, Xba I, and T4 DNA ligase. Viral DNA extraction kit was purchased from BioTeke (Beijing). SPF grade male BALB/c mice at age of 7–9 weeks and weight of 18–20 g were provided by West China Laboratory Animals Center of Sichuan University.

### 4.2. Study Procedure

#### 4.2.1. Clinical Data Collected and Analysis

Clinical data of patients who provided these serum samples were collected retrospectively from the electronic medical records in the hospital. The clinical manifestation and virological features were analyzed and compared between Han patients and Tibetan patients. Then, these data in C/D recombinant infected patients were analyzed and compared with that of all patients to learn whether the C/D recombinant had some unique clinical manifestation or virological features.

#### 4.2.2. Serum Treatment and Viral Genotype Determination

One aliquot of the collected serum sample was sent to a commercial laboratory (Kingmed Co., Sichuan) for HBV genotype determination. The laboratory used 200 *μ*L serum for extracting viral nucleic acid. Genotypes were determined by direct Sanger sequencing; the RT region of the viral genome was amplified on ABI 3130 Genetic Analyzer (South San Francisco, CA, USA) and analyzed in an alignment search tool (Chromas 2.23, Technelysium, South Brisbane, QLD, Australia) according to National Center for Biotechnology Information Genotyping Database. After acquiring the final result of genotypes, subgenotypes, or recombinants, the laboratory reported it to us. Since the C/D recombinant strain was identified, we used the other aliquot of the same serum sample for extracting viral DNA genome. In this procedure, we used the DNA extraction kit (BioTeke, Beijing). The extraction would then be used as template for PCR amplification.

#### 4.2.3. Plasmid Construction

**Figure 5:**
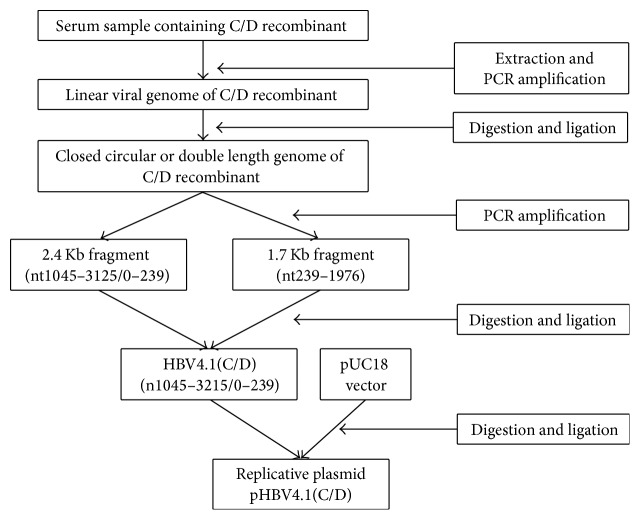
Construction procedure of clonal plasmid containing 1.3 copies of HBV genomic DNA of C/D recombinant.

Because the 1.3 copies of HBV genomic DNA could not be acquired directly, we divided the amplified DNA into two different fragments, 2.4 Kb and 1.7 Kb, at the Xba I restriction site. The two fragments both crossed the gap in the minus strand of viral genome. Thus such division would form either a circular template without gap or a linearized template with double genomic DNA. To acquire such kind of templates, enough HBV full genomic DNA need to be amplified for subsequent ligation. However, the HBV genome is nonclosed circular and partially double-strands DNA; direct amplification of the full genome is difficult. Gunther method solved this difficulty and we applied it for our study [28]. To accomplish the amplification, a primer pair (Table 3), focusing on the gap of the minus strand as well as containing specifically DR1 (direct repeat sequence 1) and the same restriction nuclease site (Sap I), was designed. The amplified DNA was then cyclized or double ligated. Then another two pairs of primers (also in Table 3) were designed to amplify the two fragments of 1.7 Kb and 2.4 Kb. Restriction sites of Hind III and Xba I were designed into the primer pair for 2.4 Kb fragment, while Xba I and Pst I were designed into the primer pair for 1.7 Kb. After digestion and gel extraction, these two fragments were ligated and inserted into the clonal plasmid vector pUC18 between the DNA restriction sites Hind III and Pst I. pUC18 is a popular vector molecule, it helped to quickly distinguish the recombinants from nonrecombinants based on colonies' color [29]. Then the monoclonal bacteria containing the recombinant plasmid were picked up for plasmid extraction. Both restriction nuclease digestion and full genome amplification with extracted plasmid were applied to confirm a successful plasmid construction. The protocol was shown in Figure 5.

#### 4.2.4. Genotype Verification and Recombinant Analysis

Direct sequencing of the amplification DNA of the extracted plasmid was used for final genotype verification. The sequencing data were input into Clustal X multiple sequence alignment program along with 18 reference genetic sequences from genotypes A to J acquired in “Basic Local Alignment Search Tool” (U.S. National Library of Medicine) for alignment. The selected reference genomic sequences were as follows: genotype A: A-1(AM282986.1) and A-2(JN182318.1); genotype B: B-1(AB368295.1) and B-2(EF494382.1); genotype C: C-1(AB368296.1) and C-2(AB368297.1); genotype D: D-1(AM422939.1) and D-2(AB554024.1); genotype E: E(FN594748.1); genotype F: F-1(AB036909.1) and F-2(AB036907.1); genotype G: G-1(AP007264.1) and G-2(AB625342.1); genotype H: H-1(AB275308.1) and H-2(AB179747.1); genotype I: I-1(FJ023659.1) and I-2(FJ023664.1); and genotype J: J(AB486012.1). Clustal X would generate an alignment file. Then the file was input into MEGA 5.10. Phylogenetic analysis was carried out in MEGA 5.10 with the Kimura two-parameter matrix model and neighboring-joining method and bootstrap resampling were performed 1,000 times. As a result, a phylogenetic tree was established and evolution of the to-be-identified HBV genome could be learned. Afterwards, the above alignment file was input into Simplot 3.5.1 to process the recombination analysis. Identity between the to-be-identified HBV genome and references sequences was calculated. The bootscan window sizes were 200 bases and the step size was 20 bases with 100 replicates [30].

#### 4.2.5. Replication Capacity Detection

Hydrodynamic HBV replicative mouse model was used to verify the replication activity of the newly constructed plasmid in vivo. A solution of 10 *μ*g naked replicative plasmid with a volume over total blood volume of a mouse (about 8% of weight) was transferred into BALB/C mouse via the tail vein within 5 to 8 seconds [26]. Circular congestion resulted in transient heart failure and backflow of the liquid from postcava to liver. Fast injection decreased the contact time of plasmid to nuclease in serum. Thus sufficient plasmid entered hepatic sinusoid and was engulfed by hepatocytes. Such mouse model provided transient viral DNA replication, RNA transcription, and protein expression lasting for 10 days with a peak around day 3. It fulfilled the requirement of studies in viral biological characteristics [31]. Besides the newly constructed plasmid; the existing plasmid pHBV4.1(D) from our lab was used as the control group. Three days after modeling, the mouse sera were collected for viral DNA extraction with the same extraction kit mentioned before. And the mouse liver was harvested to extract HBV replication intermediates.

With the same primer pair and reaction condition, full HBV genomic DNA in mouse serum was amplified. Then the amplified products were sequenced and analyzed in BLAST again to identify the genotype.

Viral replication intermediates were isolated according to previously descried methods [26]: one hundred and twenty micrograms of liver tissue in each sample was lysed for isolation and the isolated DNA replication intermediates were dissolved in 30 *μ*L 10 mmol/L tris hydrochloride (pH 8.0) and 1 mmol/L EDTA. In the protocols of HBV replication intermediates extraction, DNase was added to digest contaminated host genomic DNA and injected plasmid. We added 24 *μ*L DNase (D4527, Sigma-Aldrich, USA) solution with a concentration of 5 mg/mL to acquire a working concentration of 200 *μ*g/mL in each sample lysed from 0.12 g mouse liver powder. And with introduction of DNase digestion during the extraction procedure, the input plasmid DNA was removed and the isolated nucleotides were purified replication intermediates. DNA (Southern) filter hybridization was performed with the 30 *μ*L viral replication intermediates. Filter was probed with DIG Luminescent Detection Kit (Roche Applied Science) labeled full-length HBV genomic DNA (genotype D) and the detected replication intermediates were qualified in image analysis system (Quantity One, Bio-Rad Laboratories, Life Science). We collected Southern Blot images through an equal time interval of 5 minutes after the filter was infiltrated by luminol substrate solution. And the collection time usually lasted for 90 minutes. All these procedures were automatically implemented in ChemiDoc™ MP Imaging System (Bio-Rad, USA). Through such method, we could acquire a series of gradually enhanced images. And the proper images were picked up.

Three independent experiments were conducted, and totally six mice were modeled in each experiment: 3 in the pHBV4.1(D) group and 3 in the pHBV4.1(C/D) group.

### 4.3. Statistical Analysis

Experimental data were captured with Quantity One from photographic film. All data were analyzed with SPSS 18.00. Enumeration data were described by percentage and analyzed with *χ*^2^ test. Measurement data were described by mean ± standard deviation or median (interquartile range) according to their distribution characteristics and analyzed with *t*-test or *Z* test.

### 4.4. Ethics Statements

Experiments were performed in compliance with relevant laws and institutional guidelines and in accordance with the ethical standards of the Declaration of Helsinki. All serum samples were acquired with written informed consent under the permission from West China Hospital Ethics Committee. Animal studies were approved by Laboratory Animal Ethics Committee of Sichuan University (Project Identification Code: 2012-76).

## 5. Conclusion

In conclusion, our study found that in Sichuan Han patients from Southwest China, genotype B and C viral strains were prevalent. And this distribution was consistent with the geographic distribution of HBV genotypes in China. Meanwhile, our study found that C/D recombinant strain was rare in Han patients from Sichuan Province, yet it was dominant in Tibetan patients from Tibet. Following this finding, an HBV replicative plasmid pHBV4.1(C/D) followed by an HBV replicative mouse model was constructed. An existing plasmid pHBV4.1(D) was set as the control group mouse model. Experiment results revealed that the C/D recombinant replicated effectively at a similar level as genotype D viral strain did in the control group mouse model.

## Figures and Tables

**Table 1:** Distribution of HBV genotypes in enrolled Han patients and Tibetan patients.

Items	Statistical description percentage (amount)/median (interquartile range)		Statistical value	*p* value
	Han patients (*N* = 314)	Tibetan patients (*N* = 17)		
Genotype	B 65.92% (*N* = 207) C 30.57% (*N* = 96) B/C 2.55% (*N* = 8) C/D 0.96% (*N* = 3)	B 17.65% (*N* = 3) C 5.88% (*N* = 1) D 41.18% (*N* = 7) C/D 35.29% (*N* = 6)	Fisher	<0.001
Gender	Male 84.08% (*N* = 264) Female 15.92% (*N* = 50)	Male 82.35% (*N* = 14) Female 17.65% (*N* = 3)	*χ* ^2^ = 0.000	1
Age	33.5 (15)	38 (22.5)	*Z* = −1.019	0.308
Alcohol	Alcohol-abuse 18.47% (*N* = 58) Alcohol-free 81.53% (*N* = 256)	Alcohol-abuse 35.29% (*N* = 6) Alcohol-free 64.71% (*N* = 11)	*χ* ^2^ = 1.947	0.1629
Family history	Positive 39.17% (*N* = 123) Negative 60.83% (*N* = 191)	Positive 58.82% (*N* = 10) Negative 41.18% (*N* = 7)	*χ* ^2^ = 1.838	0.1752
ALT (IU/L)	40 (41)	38 (34.5)	*Z* = 0.286	0.388
TB (umol/L)	12.50 (9.44)	12.82 (6.76)	*Z* = −0.242	0.596
ALB (g/L)	50.70 (23.60)	47.50 (8.20)	*Z* = 1.627	0.104
ALP (IU/L)	91.50 (42)	93 (34)	*Z* = −0.213	0.831
GGT (IU/L)	49 (50)	45 (41)	*Z* = 0.483	0.629
AFP (ng/mL)	3.46 (4.25)	3.06 (5.02)	*Z* = 0.379	0.705
HBeAg	Positive 66.56% (*N* = 209) Negative 33.44% (*N* = 105)	Positive 76.47% (*N* = 13) Negative 23.53% (*N* = 4)	*χ* ^2^ = 0.33859	0.5606
log10 HBVDNA (IU/mL)	7.27 (3.21)	6.13 (1.60)	*Z* = 3.525	<0.001
BCP mutation	A1762T/G1764A 16.56% (*N* = 52) G1896A 16.56% (*N* = 52) A1762T/G1764A/G1896A 2.55% (*N* = 8) Others 3.50% (*N* = 11) Not detected 60.83% (*N* = 191)	A1762T/G1764A 41.18% (*N* = 8) G1896A 5.88% (*N* = 1) A1762T/G1764A/G1896A 5.88% (*N* = 1) Not detected 41.18% (*N* = 7)	Fisher	0.02899
Fibroscan (Kpa)	8.00 (6.20)	10.45 (5.18)	*Z* = −2.499	0.012

**Table 2:** The clinical manifestations and virological features of C/D recombinant.

Items	Statistical description percentage (amount)/median (interquartile range)		Statistical value	*p* value
	C/D recombinant infected patients (*N* = 9)	All patients (*N* = 331)		
Ethnicity	Tibetan 66.67% (*N* = 6) Han 33.33 (*N* = 3)	Tibetan 5.14% (*N* = 17) Han 94.86% (*N* = 314)	Fisher	<0.001
Gender	Male 77.78% (*N* = 7) Female 22.22% (*N* = 2)	Male 81.87% (*N* = 271) Female 18.13% (*N* = 60)	*χ* ^2^ = 0.000	1
Age	40 (22.5)	33.50 (15)	*Z* = 0.864	0.388
Alcohol	Alcohol-abuse 44.44% (*N* = 4) Alcohol-free 55.56% (*N* = 5)	Alcohol-abuse 82.48% (*N* = 273) Alcohol-free 17.52% (*N* = 58)	*χ* ^2^ = 6.0651	0.01379
Family history	Positive 77.78% (*N* = 7) Negative 22.22% (*N* = 2)	Positive 39.58% (*N* = 131) Negative 60.42% (*N* = 200)	*χ* ^2^ = 3.8364	0.05015
ALT (IU/L)	38 (32)	40 (42)	*Z* = −0.977	0.328
TB (umol/L)	11.50 (7.41)	12.40 (9.44)	*Z* = −0.611	0.542
ALB (g/L)	47.50 (9.10)	50.80 (22.40)	*Z* = −1.083	0.278
ALP (IU/L)	79 (33.25)	91 (42.25)	*Z* = −1.207	0.228
GGT (IU/L)	45.50 (34.50)	46 (54)	*Z* = −0.049	0.962
AFP (ng/mL)	3.55 (6.27)	3.42 (3.54)	*Z* = 0.069	0.944
HBeAg	Positive 77.78% (*N* = 7) Negative 22.22% (*N* = 2)	Positive 68.28% (*N* = 226) Negative 31.72% (*N* = 105)	*χ* ^2^ = 0.058456	0.809
log10 HBVDNA (IU/mL)	6.69 (1.62)	7.27 (3.04)	*Z* = −1.125	0.26
BCP mutation	A1762T/G1764A 44.44% (*N* = 4) G1896A 11.11% (*N* = 1) A1762T/G1764A/G1896A 11.11% (*N* = 1) Not detected 33.33% (*N* = 3)	A1762T/G1764A 16.01% (*N* = 53) G1896A 18.13% (*N* = 60) A1762T/G1764A/G1896A 3.02% (*N* = 10) Others 3.02% (*N* = 10) Not detected 59.82% (*N* = 198)	Fisher	0.03049
Fibroscan (Kpa)	11.5 (8.55)	7.95 (6.42)	*Z* = 1.412	0.921

**Table 3:** Primers for construction of pHBV4.1C/D with viral genome in serum sample.

Primers for amplification of HBV genome	
Upstream	5′-CCGGAAAGCTTGAGCTCTTCTTTTTCACCTCTGCCTAATCA-3′
Downstream	5′-CCGGAAAGCTTGAGCTCTTCAAAAAGTTGCATGGTGCTGG-3′

Primers for amplification of 2.4 Kb fragment	

Upstream	5′-CCTGCTTTAATGCCTTTGTATGC-3′
Downstream	5′-TCCACCACGAGTCTAGACTCTGTGG-3′

Primers for amplification of 1.7 Kb fragment	

Upstream	5′-CCACAGAGTCTAGACTCGTGGTGGA-3′
Downstream	5′-CGGTGTCGAGGAGATCTCGAATAGA-3′
